# 7α-Methoxy­carbonyl-6,7,8,14-tetra­hydro-6,14-*endo*-ethenothebaine

**DOI:** 10.1107/S1600536809009362

**Published:** 2009-03-25

**Authors:** Mustafa Odabaşoğlu, Serkan Yavuz, Özgür Pamir, Yılmaz Yıldırır, Orhan Büyükgüngör

**Affiliations:** a Chemistry Program, Denizli Higher Vocational School, Pamukkale University, TR-20159 Kınıklı, Denizli, Turkey; b Department of Chemistry, Faculty of Arts and Science, Gazi University, Ankara, Turkey; c Department of Physics, Faculty of Arts and Science, Ondokuz Mayıs University, TR-55139 Kurupelit Samsun, Turkey

## Abstract

In the mol­ecule of the title compound, C_23_H_27_NO_5_, the furan ring adopts an envelope conformation. Intra­molecular C—H⋯O inter­actions result in the formation of *S*(5) and *S*(6) motifs. In the crystal structure, weak inter­molecular C—H⋯O hydrogen bonds link the mol­ecules through *C*(6) and *C*(8) chains along the [100] and [010] directions, generating a two-dimensional network.

## Related literature

For general background, see: Casy & Parfitt (1986 ▶); Lenz *et al.* (1986 ▶); Schmidhammer, (1998 ▶); Maat *et al.* (1999 ▶); Lewis (1985 ▶). For a related structure, see: Bentley & Hardy (1967 ▶). For bond-length data, see: Allen *et al.* (1987 ▶). For ring-puckering parameters, see: Cremer & Pople (1975 ▶). For ring motifs, see: Bernstein *et al.* (1995 ▶); Etter (1990 ▶).
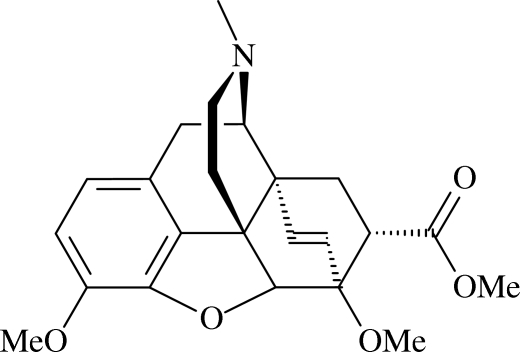


## Experimental

### 

#### Crystal data

C_23_H_27_NO_5_
*M* *_r_* = 397.46
Orthorhombic, 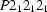

*a* = 6.5604 (2) Å
*b* = 10.4082 (3) Å
*c* = 29.1382 (11) Å
*V* = 1989.61 (11) Å^3^
*Z* = 4
Mo *K*α radiation
μ = 0.09 mm^−1^
*T* = 296 K
0.39 × 0.35 × 0.31 mm

#### Data collection

Stoe IPDS II diffractometer
Absorption correction: integration (*X-RED32*; Stoe & Cie, 2002 ▶) *T*_min_ = 0.966, *T*_max_ = 0.984
27330 measured reflections
2467 independent reflections
2300 reflections with *I* > 2σ(*I*)
*R*_int_ = 0.037

#### Refinement

*R*[*F* ^2^ > 2σ(*F* ^2^)] = 0.032
*wR*(*F* ^2^) = 0.085
*S* = 1.06
2467 reflections
262 parameters
H-atom parameters constrained
Δρ_max_ = 0.14 e Å^−3^
Δρ_min_ = −0.14 e Å^−3^

### 

Data collection: *X-AREA* (Stoe & Cie, 2002 ▶); cell refinement: *X-AREA*; data reduction: *X-RED32* (Stoe & Cie, 2002 ▶); program(s) used to solve structure: *SHELXS97* (Sheldrick, 2008 ▶); program(s) used to refine structure: *SHELXL97* (Sheldrick, 2008 ▶); molecular graphics: *ORTEP-3 for Windows* (Farrugia, 1997 ▶); software used to prepare material for publication: *WinGX* (Farrugia, 1999 ▶).

## Supplementary Material

Crystal structure: contains datablocks I, global. DOI: 10.1107/S1600536809009362/hk2636sup1.cif

Structure factors: contains datablocks I. DOI: 10.1107/S1600536809009362/hk2636Isup2.hkl

Additional supplementary materials:  crystallographic information; 3D view; checkCIF report

## Figures and Tables

**Table 1:** Hydrogen-bond geometry (Å, °)

*D*—H⋯*A*	*D*—H	H⋯*A*	*D*⋯*A*	*D*—H⋯*A*
C7—H7*A*⋯O2	0.96	2.34	3.002 (3)	125
C12—H12*B*⋯O4	0.97	2.46	2.899 (2)	107
C15—H15⋯O4^i^	0.98	2.53	3.483 (2)	165
C17—H17*C*⋯O2^ii^	0.96	2.62	3.533 (3)	158

Symmetry codes: (i) 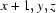; (ii) 
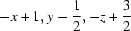
.

**Fig. 1.**
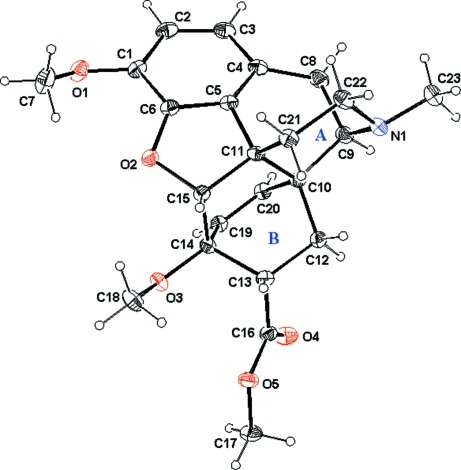
The molecular structure of the title molecule, with the atom-numbering scheme. Displacement ellipsoids are drawn at the 20% probability level.

**Fig. 2.**
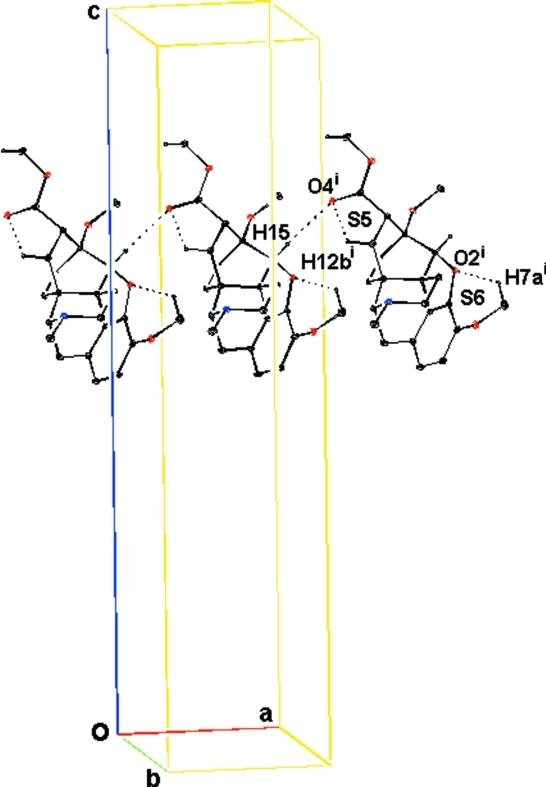
A partial packing diagram of the title compound, showing the formation of C(6) chain with S(5) and S(6) motifs along the [100] direction. H atoms not involved in hydrogen bonding have been omitted. Hydrogen bonds are shown as dashed lines [symmetry code: (i) x + 1, y, z].

**Fig. 3.**
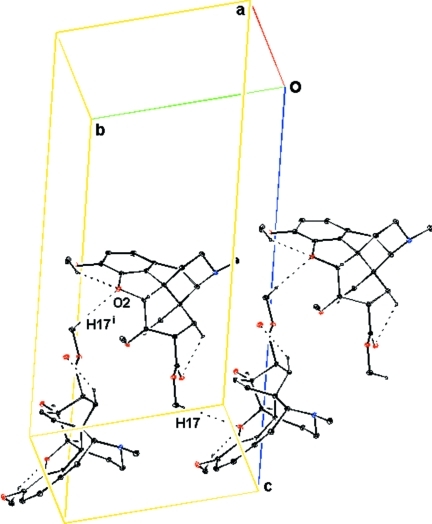
A partial packing diagram of the title compound, showing the formation of C(8) chain along the [010] direction. H atoms not involved in hydrogen bonding have been omitted. Hydrogen bonds are shown as dashed lines [symmetry code: (i) x - 1, y + 1/2, z].

**Fig. 4.**
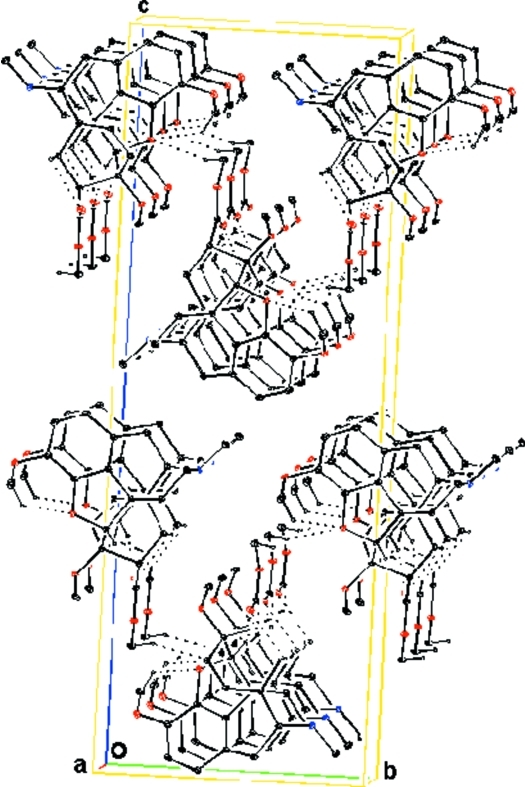
A partial packing diagram of the title compound. Hydrogen bonds are shown as dashed lines. H atoms not involved in hydrogen bonding have been omitted.

## supplementary crystallographic information

### Comment

Morphine alkaloids and related semisynthetic derivatives are the most important groups of non-endogenous opioid-receptor ligands. They can possess both agonist and antagonist properties. Thus, some of them are used as effective analgesics for the treatment of moderate to severe pain or as opioid antagonists for the treatment of narcotic overdosage or opioid addiction; others are used as intermediate products in research (Casy & Parfitt, 1986; Lenz *et al.*,  1986; Schmidhammer, 1998).

Thebaine readily undergoes Diels–Alder reactions with various dienophiles to give the adducts. The diene system of thebaine could potentially be attacked from both faces, but reactions with dienophiles always occur from the same face as the nitrogen bridge (upper face) due to the nitrogen bridge causing the lower face to be hindered through concealment inside a concave system (Maat *et al.*,  1999). The nature of a substituent in positions 7,8 of morphinane alkaloids is among the most important factors affecting their biological activity (Lenz *et al.*,  1986). For instance, the opioid analgesic buprenorphine, ethorphine possesses a pharmacological profile interesting for development of antinarcotics (Lewis, 1985). In view of the importance of the morphine alkaloids, we report herein the crystal structure of the title compound.

In the molecule of the title compound (Fig. 1), the bond lengths (Allen *et al.*,  1987) and angles are within normal ranges. Rings C (C1–C6), D (O2/C5/C6/C11/C15) and E (C4/C5/C8–C11) adopt envelope conformations with C5, C15 and C10 atoms displaced by 0.118 (3), -0.172 (3) and -0.809 (3) Å from the planes of the other ring atoms, respectively. Rings A (N1/C9–C11/C21/C22), B (C10/C12–C14/C19/C20), F (C10–C15) and G (C10/C11/C14/C15/C19/C20) are not planar, having total puckering amplitudes, Q_T_, of 0.604 (2), 0.820 (2), 1.047 (2) and 0.942 Å and chair, boat, boat and boat conformations [φ = 78.15 (3) and θ = 9.43 (3)° (for ring A), φ = 3.08 (3) and θ = 87.76 (3)° (for ring B), φ = 155.88 (3) and θ = 152.64 (3)° (for ring F) and φ = -82.44 (3) and θ = 148.46 (3)° (for ring G)] (Cremer & Pople, 1975). The intramolecular C—H···O interactions (Table 1) result in the formations of five- and six-membered rings H (O4/C12/C13/C16/H12B) and I (O1/O2/C1/C6/C7/H7A). Ring H adopts envelope conformation with O4 atom displaced by 0.536 (4) Å from the plane of the other ring atoms, while ring I has twisted conformation. The intramolecular interactions result in the formations of S(5) and S(6) motifs (Bernstein *et al.*,  1995; Etter, 1990) (Fig. 2).

In the crystal structure, weak intermolecular C—H···O hydrogen bonds (Table 1) link the molecules through C(6) and C(8) chains (Bernstein *et al.*,  1995; Etter, 1990) nearly along the [100] and [010] directions (Figs. 2 and 3), generating a three-dimensional network (Fig. 4).

### Experimental

The title compound was prepared according to the literature method (Bentley & Hardy, 1967). Thebaine (1.50 g, 4.82 mmol) and methyl acrylate (2.55 ml, 28.3 mmol) were refluxed in benzene (50 ml) for 8 h. After cooling to room temperature, the reaction mixture was concentrated *in vacuo*. The mixture was centrifuged, and then the title compound was obtained as colorless prisms. It was washed with cold methanol, and recrystallized from methanol.

### Refinement

H atoms were positioned geometrically, with C—H = 0.93, 0.98, 0.97 and 0.96 Å for aromatic, methine, methylene and methyl H, respectively, and constrained to ride on their parent atoms, with U_iso_(H) = 1.2U_eq_(C). The absolute structure could not be determined reliably, and 1795 Friedel pairs were averaged before the last cycle of refinement.

### Crystal data

**Table:**

C_23_H_27_NO_5_	*F*(000) = 848
*M**_r_* = 397.46	*D*_x_ = 1.327 Mg m^−^^3^
Orthorhombic, *P*2_1_2_1_2_1_	Mo *K*α radiation, λ = 0.71073 Å
Hall symbol: P 2ac 2ab	Cell parameters from 27330 reflections
*a* = 6.5604 (2) Å	θ = 1.4–27.3°
*b* = 10.4082 (3) Å	µ = 0.09 mm^−^^1^
*c* = 29.1382 (11) Å	*T* = 296 K
*V* = 1989.61 (11) Å^3^	Prism, colourless
*Z* = 4	0.39 × 0.35 × 0.31 mm

### Data collection

**Table:**

Stoe IPDS II diffractometer	2467 independent reflections
Radiation source: fine-focus sealed tube	2300 reflections with *I* > 2σ(*I*)
graphite	*R*_int_ = 0.037
ω scan rotation method	θ_max_ = 26.8°, θ_min_ = 1.4°
Absorption correction: integration (*X-RED32*; Stoe & Cie, 2002)	*h* = −8→8
*T*_min_ = 0.966, *T*_max_ = 0.984	*k* = −12→13
27330 measured reflections	*l* = −36→36

### Refinement

**Table:**

Refinement on *F*^2^	Primary atom site location: structure-invariant direct methods
Least-squares matrix: full	Secondary atom site location: difference Fourier map
*R*[*F*^2^ > 2σ(*F*^2^)] = 0.032	Hydrogen site location: inferred from neighbouring sites
*wR*(*F*^2^) = 0.085	H-atom parameters constrained
*S* = 1.06	*w* = 1/[σ^2^(*F*_o_^2^) + (0.0516*P*)^2^ + 0.2082*P*] where *P* = (*F*_o_^2^ + 2*F*_c_^2^)/3
2467 reflections	(Δ/σ)_max_ = 0.001
262 parameters	Δρ_max_ = 0.14 e Å^−^^3^
0 restraints	Δρ_min_ = −0.14 e Å^−^^3^

### Special details

**Table:**

Geometry. All e.s.d.'s (except the e.s.d. in the dihedral angle between two l.s. planes) are estimated using the full covariance matrix. The cell e.s.d.'s are taken into account individually in the estimation of e.s.d.'s in distances, angles and torsion angles; correlations between e.s.d.'s in cell parameters are only used when they are defined by crystal symmetry. An approximate (isotropic) treatment of cell e.s.d.'s is used for estimating e.s.d.'s involving l.s. planes.
Refinement. Refinement of *F*^2^ against ALL reflections. The weighted *R*-factor *wR* and goodness of fit *S* are based on *F*^2^, conventional *R*-factors *R* are based on *F*, with *F* set to zero for negative *F*^2^. The threshold expression of *F*^2^ > σ(*F*^2^) is used only for calculating *R*-factors(gt) *etc*. and is not relevant to the choice of reflections for refinement. *R*-factors based on *F*^2^ are statistically about twice as large as those based on *F*, and *R*- factors based on ALL data will be even larger.

### Fractional atomic coordinates and isotropic or equivalent isotropic displacement parameters (Å^2^)

**Table:**

	*x*	*y*	*z*	*U*_iso_*/*U*_eq_	
O1	0.9853 (3)	0.82364 (14)	0.58760 (5)	0.0549 (4)	
O2	0.9330 (2)	0.60075 (12)	0.65159 (4)	0.0386 (3)	
O3	0.6842 (2)	0.59127 (12)	0.73570 (4)	0.0395 (3)	
O4	0.2507 (2)	0.38987 (17)	0.74242 (5)	0.0535 (4)	
O5	0.4979 (2)	0.37509 (14)	0.79459 (4)	0.0460 (3)	
N1	0.6584 (3)	0.16016 (16)	0.58745 (5)	0.0444 (4)	
C1	0.9098 (3)	0.70471 (19)	0.57577 (7)	0.0435 (5)	
C2	0.8226 (4)	0.6904 (2)	0.53244 (7)	0.0521 (5)	
H2	0.8344	0.7572	0.5114	0.063*	
C3	0.7190 (4)	0.5803 (2)	0.51963 (6)	0.0503 (5)	
H3	0.6560	0.5766	0.4911	0.060*	
C4	0.7078 (3)	0.47442 (19)	0.54914 (6)	0.0415 (4)	
C5	0.8120 (3)	0.48664 (17)	0.59018 (6)	0.0353 (4)	
C6	0.8950 (3)	0.60011 (19)	0.60527 (6)	0.0375 (4)	
C7	1.1676 (5)	0.8241 (3)	0.61339 (10)	0.0721 (7)	
H7A	1.1464	0.7790	0.6417	0.087*	
H7B	1.2068	0.9112	0.6198	0.087*	
H7C	1.2735	0.7825	0.5962	0.087*	
C8	0.5653 (4)	0.3625 (2)	0.54286 (6)	0.0484 (5)	
H8A	0.6178	0.3088	0.5184	0.058*	
H8B	0.4341	0.3953	0.5330	0.058*	
C9	0.5316 (3)	0.27660 (19)	0.58627 (6)	0.0410 (4)	
H9	0.3887	0.2491	0.5862	0.049*	
C10	0.5680 (3)	0.35311 (17)	0.63073 (6)	0.0337 (4)	
C11	0.7950 (3)	0.39378 (17)	0.62929 (6)	0.0328 (4)	
C12	0.5280 (3)	0.28000 (17)	0.67567 (6)	0.0385 (4)	
H12A	0.6025	0.1995	0.6755	0.046*	
H12B	0.3838	0.2607	0.6784	0.046*	
C13	0.5970 (3)	0.36300 (17)	0.71682 (6)	0.0346 (4)	
H13	0.7151	0.3225	0.7315	0.042*	
C14	0.6587 (3)	0.50225 (16)	0.69954 (5)	0.0325 (4)	
C15	0.8520 (3)	0.48032 (16)	0.67040 (6)	0.0320 (4)	
H15	0.9566	0.4380	0.6890	0.038*	
C16	0.4276 (3)	0.37757 (17)	0.75171 (6)	0.0373 (4)	
C17	0.3443 (4)	0.3871 (2)	0.82992 (7)	0.0582 (6)	
H17A	0.2733	0.4671	0.8262	0.070*	
H17B	0.4082	0.3850	0.8595	0.070*	
H17C	0.2493	0.3173	0.8274	0.070*	
C18	0.8627 (4)	0.5760 (2)	0.76315 (7)	0.0531 (5)	
H18A	0.8614	0.4926	0.7772	0.064*	
H18B	0.8652	0.6409	0.7865	0.064*	
H18C	0.9815	0.5841	0.7441	0.064*	
C19	0.4885 (3)	0.54957 (17)	0.66960 (6)	0.0348 (4)	
H19	0.4201	0.6261	0.6754	0.042*	
C20	0.4425 (3)	0.47512 (18)	0.63434 (6)	0.0366 (4)	
H20	0.3410	0.4960	0.6133	0.044*	
C21	0.9344 (3)	0.27647 (18)	0.62531 (6)	0.0398 (4)	
H21A	1.0739	0.3051	0.6211	0.048*	
H21B	0.9282	0.2277	0.6536	0.048*	
C22	0.8753 (3)	0.1899 (2)	0.58551 (7)	0.0478 (5)	
H22A	0.9064	0.2324	0.5567	0.057*	
H22B	0.9535	0.1109	0.5869	0.057*	
C23	0.5999 (4)	0.0659 (2)	0.55288 (8)	0.0594 (6)	
H23A	0.4611	0.0401	0.5578	0.071*	
H23B	0.6873	−0.0078	0.5551	0.071*	
H23C	0.6127	0.1032	0.5229	0.071*	

### Atomic displacement parameters (Å^2^)

**Table:**

	*U*^11^	*U*^22^	*U*^33^	*U*^12^	*U*^13^	*U*^23^
O1	0.0602 (10)	0.0446 (7)	0.0601 (9)	−0.0062 (7)	−0.0022 (8)	0.0136 (7)
O2	0.0409 (7)	0.0403 (7)	0.0345 (6)	−0.0067 (6)	−0.0023 (5)	0.0046 (5)
O3	0.0414 (7)	0.0397 (7)	0.0374 (6)	0.0075 (6)	−0.0061 (6)	−0.0074 (5)
O4	0.0400 (8)	0.0721 (10)	0.0485 (7)	0.0135 (7)	0.0066 (6)	0.0086 (7)
O5	0.0502 (8)	0.0562 (8)	0.0317 (6)	−0.0020 (7)	0.0068 (6)	−0.0015 (6)
N1	0.0515 (10)	0.0427 (9)	0.0390 (8)	−0.0005 (8)	0.0022 (8)	−0.0086 (7)
C1	0.0426 (11)	0.0426 (10)	0.0454 (10)	−0.0006 (9)	0.0052 (8)	0.0082 (8)
C2	0.0590 (13)	0.0576 (12)	0.0398 (10)	0.0047 (12)	0.0044 (10)	0.0176 (9)
C3	0.0566 (12)	0.0632 (13)	0.0310 (8)	0.0078 (11)	−0.0025 (9)	0.0083 (8)
C4	0.0438 (11)	0.0509 (11)	0.0299 (8)	0.0059 (9)	−0.0008 (8)	0.0013 (8)
C5	0.0328 (9)	0.0427 (9)	0.0305 (8)	0.0023 (8)	0.0022 (7)	0.0042 (7)
C6	0.0323 (9)	0.0463 (10)	0.0338 (8)	0.0017 (8)	0.0015 (7)	0.0046 (7)
C7	0.0646 (16)	0.0618 (15)	0.0900 (19)	−0.0224 (14)	−0.0152 (15)	0.0162 (14)
C8	0.0557 (12)	0.0555 (12)	0.0339 (9)	0.0004 (11)	−0.0091 (9)	−0.0025 (8)
C9	0.0395 (10)	0.0471 (10)	0.0363 (9)	−0.0033 (8)	−0.0033 (8)	−0.0044 (8)
C10	0.0321 (8)	0.0382 (9)	0.0307 (8)	−0.0007 (7)	0.0004 (7)	−0.0010 (7)
C11	0.0330 (9)	0.0383 (9)	0.0271 (7)	0.0032 (7)	−0.0010 (7)	0.0019 (7)
C12	0.0416 (10)	0.0356 (9)	0.0384 (9)	−0.0028 (8)	0.0065 (8)	−0.0018 (7)
C13	0.0359 (9)	0.0366 (9)	0.0313 (8)	0.0051 (8)	0.0026 (7)	0.0025 (7)
C14	0.0361 (9)	0.0328 (8)	0.0287 (8)	0.0028 (7)	−0.0010 (7)	−0.0011 (6)
C15	0.0305 (8)	0.0344 (8)	0.0311 (8)	0.0007 (7)	−0.0032 (7)	0.0034 (7)
C16	0.0424 (10)	0.0347 (9)	0.0347 (9)	0.0030 (8)	0.0044 (8)	0.0033 (7)
C17	0.0666 (15)	0.0672 (14)	0.0408 (10)	−0.0003 (13)	0.0186 (11)	−0.0031 (10)
C18	0.0519 (12)	0.0616 (13)	0.0457 (10)	0.0102 (11)	−0.0151 (10)	−0.0155 (9)
C19	0.0326 (9)	0.0346 (8)	0.0372 (9)	0.0050 (7)	0.0003 (7)	0.0027 (7)
C20	0.0296 (9)	0.0433 (9)	0.0370 (8)	0.0018 (8)	−0.0030 (7)	0.0037 (8)
C21	0.0350 (9)	0.0441 (9)	0.0403 (9)	0.0059 (8)	−0.0006 (8)	−0.0020 (8)
C22	0.0493 (12)	0.0492 (11)	0.0449 (10)	0.0089 (9)	0.0036 (9)	−0.0088 (9)
C23	0.0730 (16)	0.0523 (13)	0.0527 (12)	−0.0053 (12)	0.0027 (12)	−0.0173 (10)

### Geometric parameters (Å, °)

**Table:**

C1—O1	1.377 (3)	C13—C14	1.587 (2)
C1—C6	1.390 (3)	C13—H13	0.9800
C1—C2	1.394 (3)	C14—O3	1.413 (2)
C2—C3	1.383 (3)	C14—C19	1.500 (2)
C2—H2	0.9300	C14—C15	1.542 (2)
C3—C4	1.400 (3)	C15—O2	1.468 (2)
C3—H3	0.9300	C15—H15	0.9800
C4—C5	1.383 (2)	C16—O4	1.199 (2)
C4—C8	1.505 (3)	C16—O5	1.332 (2)
C5—C6	1.373 (3)	C17—O5	1.446 (2)
C5—C11	1.499 (2)	C17—H17A	0.9600
C6—O2	1.373 (2)	C17—H17B	0.9600
C7—O1	1.412 (3)	C17—H17C	0.9600
C7—H7A	0.9600	C18—O3	1.427 (2)
C7—H7B	0.9600	C18—H18A	0.9600
C7—H7C	0.9600	C18—H18B	0.9600
C8—C9	1.565 (3)	C18—H18C	0.9600
C8—H8A	0.9700	C19—C20	1.322 (3)
C8—H8B	0.9700	C19—H19	0.9300
C9—N1	1.470 (3)	C20—H20	0.9300
C9—C10	1.539 (2)	C21—C22	1.519 (3)
C9—H9	0.9800	C21—H21A	0.9700
C10—C20	1.517 (2)	C21—H21B	0.9700
C10—C12	1.537 (2)	C22—N1	1.457 (3)
C10—C11	1.549 (3)	C22—H22A	0.9700
C11—C21	1.530 (2)	C22—H22B	0.9700
C11—C15	1.545 (2)	C23—N1	1.458 (3)
C12—C13	1.546 (2)	C23—H23A	0.9600
C12—H12A	0.9700	C23—H23B	0.9600
C12—H12B	0.9700	C23—H23C	0.9600
C13—C16	1.514 (2)		
			
O1—C1—C6	125.05 (18)	C14—C13—H13	109.3
O1—C1—C2	118.06 (18)	O3—C14—C19	107.84 (14)
C6—C1—C2	116.58 (19)	O3—C14—C15	114.23 (15)
C3—C2—C1	122.32 (18)	C19—C14—C15	109.89 (13)
C3—C2—H2	118.8	O3—C14—C13	113.10 (13)
C1—C2—H2	118.8	C19—C14—C13	107.12 (15)
C2—C3—C4	120.79 (18)	C15—C14—C13	104.43 (13)
C2—C3—H3	119.6	O2—C15—C14	112.14 (13)
C4—C3—H3	119.6	O2—C15—C11	107.22 (13)
C5—C4—C3	115.65 (19)	C14—C15—C11	108.33 (14)
C5—C4—C8	118.90 (16)	O2—C15—H15	109.7
C3—C4—C8	124.56 (17)	C14—C15—H15	109.7
C6—C5—C4	123.50 (17)	C11—C15—H15	109.7
C6—C5—C11	109.92 (15)	O4—C16—O5	123.30 (18)
C4—C5—C11	124.16 (17)	O4—C16—C13	124.74 (17)
O2—C6—C5	113.03 (16)	O5—C16—C13	111.95 (16)
O2—C6—C1	126.25 (18)	O5—C17—H17A	109.5
C5—C6—C1	120.22 (17)	O5—C17—H17B	109.5
O1—C7—H7A	109.5	H17A—C17—H17B	109.5
O1—C7—H7B	109.5	O5—C17—H17C	109.5
H7A—C7—H7B	109.5	H17A—C17—H17C	109.5
O1—C7—H7C	109.5	H17B—C17—H17C	109.5
H7A—C7—H7C	109.5	O3—C18—H18A	109.5
H7B—C7—H7C	109.5	O3—C18—H18B	109.5
C4—C8—C9	115.59 (15)	H18A—C18—H18B	109.5
C4—C8—H8A	108.4	O3—C18—H18C	109.5
C9—C8—H8A	108.4	H18A—C18—H18C	109.5
C4—C8—H8B	108.4	H18B—C18—H18C	109.5
C9—C8—H8B	108.4	C20—C19—C14	115.44 (16)
H8A—C8—H8B	107.4	C20—C19—H19	122.3
N1—C9—C10	108.61 (15)	C14—C19—H19	122.3
N1—C9—C8	114.22 (17)	C19—C20—C10	114.87 (16)
C10—C9—C8	111.27 (15)	C19—C20—H20	122.6
N1—C9—H9	107.5	C10—C20—H20	122.6
C10—C9—H9	107.5	C22—C21—C11	112.26 (16)
C8—C9—H9	107.5	C22—C21—H21A	109.2
C20—C10—C12	105.24 (14)	C11—C21—H21A	109.2
C20—C10—C9	114.03 (15)	C22—C21—H21B	109.2
C12—C10—C9	115.75 (15)	C11—C21—H21B	109.2
C20—C10—C11	107.15 (14)	H21A—C21—H21B	107.9
C12—C10—C11	108.81 (15)	N1—C22—C21	110.23 (17)
C9—C10—C11	105.52 (15)	N1—C22—H22A	109.6
C5—C11—C21	114.36 (15)	C21—C22—H22A	109.6
C5—C11—C15	101.29 (14)	N1—C22—H22B	109.6
C21—C11—C15	112.32 (14)	C21—C22—H22B	109.6
C5—C11—C10	105.56 (14)	H22A—C22—H22B	108.1
C21—C11—C10	111.03 (15)	N1—C23—H23A	109.5
C15—C11—C10	111.77 (14)	N1—C23—H23B	109.5
C10—C12—C13	109.52 (14)	H23A—C23—H23B	109.5
C10—C12—H12A	109.8	N1—C23—H23C	109.5
C13—C12—H12A	109.8	H23A—C23—H23C	109.5
C10—C12—H12B	109.8	H23B—C23—H23C	109.5
C13—C12—H12B	109.8	C22—N1—C23	111.90 (18)
H12A—C12—H12B	108.2	C22—N1—C9	112.11 (17)
C16—C13—C12	111.21 (16)	C23—N1—C9	112.95 (17)
C16—C13—C14	107.99 (14)	C1—O1—C7	116.17 (18)
C12—C13—C14	109.82 (13)	C6—O2—C15	107.29 (14)
C16—C13—H13	109.3	C14—O3—C18	116.21 (15)
C12—C13—H13	109.3	C16—O5—C17	115.13 (17)
			
O1—C1—C2—C3	−171.3 (2)	C16—C13—C14—C19	−72.07 (17)
C6—C1—C2—C3	2.5 (3)	C12—C13—C14—C19	49.36 (19)
C1—C2—C3—C4	−3.8 (4)	C16—C13—C14—C15	171.38 (14)
C2—C3—C4—C5	−2.2 (3)	C12—C13—C14—C15	−67.18 (17)
C2—C3—C4—C8	166.8 (2)	O3—C14—C15—O2	−55.46 (18)
C3—C4—C5—C6	10.0 (3)	C19—C14—C15—O2	65.87 (18)
C8—C4—C5—C6	−159.66 (19)	C13—C14—C15—O2	−179.52 (13)
C3—C4—C5—C11	170.57 (18)	O3—C14—C15—C11	−173.59 (13)
C8—C4—C5—C11	0.9 (3)	C19—C14—C15—C11	−52.26 (18)
C4—C5—C6—O2	160.73 (17)	C13—C14—C15—C11	62.35 (16)
C11—C5—C6—O2	−2.2 (2)	C5—C11—C15—O2	−10.94 (17)
C4—C5—C6—C1	−11.7 (3)	C21—C11—C15—O2	111.52 (16)
C11—C5—C6—C1	−174.64 (17)	C10—C11—C15—O2	−122.92 (14)
O1—C1—C6—O2	6.9 (3)	C5—C11—C15—C14	110.28 (15)
C2—C1—C6—O2	−166.41 (19)	C21—C11—C15—C14	−127.26 (15)
O1—C1—C6—C5	178.25 (19)	C10—C11—C15—C14	−1.70 (18)
C2—C1—C6—C5	4.9 (3)	C12—C13—C16—O4	−37.8 (3)
C5—C4—C8—C9	6.1 (3)	C14—C13—C16—O4	82.8 (2)
C3—C4—C8—C9	−162.6 (2)	C12—C13—C16—O5	142.17 (16)
C4—C8—C9—N1	−97.5 (2)	C14—C13—C16—O5	−97.26 (17)
C4—C8—C9—C10	25.9 (3)	O3—C14—C19—C20	−178.76 (16)
N1—C9—C10—C20	−178.89 (16)	C15—C14—C19—C20	56.2 (2)
C8—C9—C10—C20	54.6 (2)	C13—C14—C19—C20	−56.72 (19)
N1—C9—C10—C12	−56.6 (2)	C14—C19—C20—C10	1.0 (2)
C8—C9—C10—C12	176.87 (18)	C12—C10—C20—C19	59.6 (2)
N1—C9—C10—C11	63.79 (18)	C9—C10—C20—C19	−172.53 (17)
C8—C9—C10—C11	−62.8 (2)	C11—C10—C20—C19	−56.2 (2)
C6—C5—C11—C21	−112.91 (18)	C5—C11—C21—C22	−66.6 (2)
C4—C5—C11—C21	84.3 (2)	C15—C11—C21—C22	178.70 (16)
C6—C5—C11—C15	8.13 (19)	C10—C11—C21—C22	52.7 (2)
C4—C5—C11—C15	−154.69 (18)	C11—C21—C22—N1	−50.8 (2)
C6—C5—C11—C10	124.75 (16)	C21—C22—N1—C23	−173.61 (17)
C4—C5—C11—C10	−38.1 (2)	C21—C22—N1—C9	58.3 (2)
C20—C10—C11—C5	−55.15 (18)	C10—C9—N1—C22	−66.6 (2)
C12—C10—C11—C5	−168.46 (14)	C8—C9—N1—C22	58.2 (2)
C9—C10—C11—C5	66.73 (17)	C10—C9—N1—C23	165.85 (17)
C20—C10—C11—C21	−179.60 (13)	C8—C9—N1—C23	−69.3 (2)
C12—C10—C11—C21	67.09 (18)	C6—C1—O1—C7	45.0 (3)
C9—C10—C11—C21	−57.73 (18)	C2—C1—O1—C7	−141.8 (2)
C20—C10—C11—C15	54.12 (18)	C5—C6—O2—C15	−5.3 (2)
C12—C10—C11—C15	−59.19 (17)	C1—C6—O2—C15	166.61 (18)
C9—C10—C11—C15	175.99 (14)	C14—C15—O2—C6	−108.49 (15)
C20—C10—C12—C13	−60.31 (19)	C11—C15—O2—C6	10.29 (18)
C9—C10—C12—C13	172.82 (16)	C19—C14—O3—C18	−168.91 (17)
C11—C10—C12—C13	54.25 (19)	C15—C14—O3—C18	−46.5 (2)
C10—C12—C13—C16	126.85 (16)	C13—C14—O3—C18	72.8 (2)
C10—C12—C13—C14	7.4 (2)	O4—C16—O5—C17	0.6 (3)
C16—C13—C14—O3	46.6 (2)	C13—C16—O5—C17	−179.37 (16)
C12—C13—C14—O3	168.04 (15)		

### Hydrogen-bond geometry (Å, °)

**Table:**

*D*—H···*A*	*D*—H	H···*A*	*D*···*A*	*D*—H···*A*
C7—H7A···O2	0.96	2.34	3.002 (3)	125
C12—H12B···O4	0.97	2.46	2.899 (2)	107
C15—H15···O4^i^	0.98	2.53	3.483 (2)	165
C17—H17C···O2^ii^	0.96	2.62	3.533 (3)	158

Symmetry codes: (i) *x*+1, *y*, *z*; (ii) −*x*+1, *y*−1/2, −*z*+3/2.
